# Development of the Novel Bifunctional Fusion Protein BR102 That Simultaneously Targets PD-L1 and TGF-β for Anticancer Immunotherapy

**DOI:** 10.3390/cancers14194964

**Published:** 2022-10-10

**Authors:** Zhen-Hua Wu, Na Li, Zhang-Zhao Gao, Gang Chen, Lei Nie, Ya-Qiong Zhou, Mei-Zhu Jiang, Yao Chen, Juan Chen, Xiao-Fen Mei, Feng Hu, Hai-Bin Wang

**Affiliations:** 1 BioRay Pharmaceutical Co., Ltd., Taizhou 318000, China; 2 BioRay Pharmaceutical Corp, San Diego, CA 92121, USA

**Keywords:** cancer immunotherapy, PD-L1, TGF-β, bifunctional fusion protein, BR102

## Abstract

**Simple Summary:**

Immune checkpoint inhibitors (ICIs), such as anti-PD-1/PD-L1 antibodies, have revolutionized the therapy landscape of cancer immunotherapy. However, poor clinical response to ICIs and drug resistance are the main challenges for ICIs immunotherapy. TGF-β produced in the TME was found to confer resistance to PD-1/PD-L1-targeted immunotherapy. The independent and complementary immunosuppressive role of PD-L1 and TGF-β in cancer progression provides a rationale for simultaneously targeting TGF-β and PD-L1 to improve anti-PD-L1 therapy. Consequently, we develop and characterize a novel anti-PD-L1/TGF-β bifunctional fusion protein termed BR102. The data suggest that BR102 could simultaneously disrupt TGF-β- and PD-L1-mediated signals and display high antitumor efficacy and safety. The data support further clinical advancement of BR102 as a promising approach to cancer immunotherapy.

**Abstract:**

Immune checkpoint inhibitors (ICIs) are remarkable breakthroughs in treating various types of cancer, but many patients still do not derive long-term clinical benefits. Increasing evidence shows that TGF-β can promote cancer progression and confer resistance to ICI therapies. Consequently, dual blocking of TGF-β and immune checkpoint may provide an effective approach to enhance the effectiveness of ICI therapies. Here, we reported the development and preclinical characterization of a novel bifunctional anti-PD-L1/TGF-β fusion protein, BR102. BR102 comprises an anti-PD-L1 antibody fused to the extracellular domain (ECD) of human TGF-βRII. BR102 is capable of simultaneously binding to TGF-β and PD-L1. Incorporating TGF-βRII into BR102 does not alter the PD-L1 blocking activity of BR102. In vitro characterization further demonstrated that BR102 could disrupt TGF-β-induced signaling. Moreover, BR102 significantly inhibits tumor growth in vivo and exerts a superior antitumor effect compared to anti-PD-L1. Administration of BR102 to cynomolgus monkeys is well-tolerated, with only minimal to moderate and reversing red cell changes noted. The data demonstrated the efficacy and safety of the novel anti-PD-L1/TGF-β fusion protein and supported the further clinical development of BR102 for anticancer therapy.

## 1. Introduction

Immune checkpoint molecules, including PD-1/PD-L1, CTLA-4, and LAG-3, play significant roles in mediating T-cell dysfunction during cancer progression and suppressing antitumor immunity. Cancer immunotherapy targeting immune checkpoint molecules can restore antitumor immune response to kill tumor cells. ICIs, such as monoclonal antibodies against PD-1, PD-L1 and CTLA-4, have greatly improved the clinical outcome of cancer patients [1,2], revolutionizing the treatment landscape for multiple tumor types. However, many cancer patients achieve only a short-lived clinical benefit from checkpoint blockade therapies, and some may eventually develop resistance which leads to disease progression [3,4,5]. This highlights the urgent need for alternative approaches to improve response rates.

TGF-β is a multifunctional cytokine involved in many developmental and metabolic processes, including proliferation, differentiation, apoptosis, angiogenesis, and cellular immune response [6]. It has three highly-conserved isoforms: TGF-β1, TGF-β2, and TGF-β3, that interact with a tetrameric receptor complex to transmit intracellular signaling. TGF-β signaling activation may lead to different effects in a context-dependent manner. In healthy and pre-malignant cells, TGF-β functions as a tumor suppressor by promoting apoptosis and cell-cycle arrest. However, cancer cells can bypass the suppressor effects of TGF-β and subvert TGF-β activity to obtain a growth advantage. TGF-β produced in the TME induces epithelial-to-mesenchymal transition (EMT), which drives migration and invasion of tumor cells [7,8]. Moreover, TGF-β also contributes to tumor progression by increasing extracellular matrix (ECM) production, activating cancer-associated fibroblasts (CAFs), promoting angiogenesis, and stimulating immune evasion [9]. High TGF-β expression correlates with poor prognosis in several tumor types [10,11]. Based on the preclinical findings that targeting TGF-β could exert antitumor activity, many pharmacological TGF-β inhibitors have been discovered and evaluated in clinical trials, including receptor kinases inhibitors, neutralizing antibodies, antisense oligonucleotides, and ligand traps. However, the tumor suppressive activity of TGF-β and its pleiotropic nature prevented rapid clinical translation of anti-TGF-β therapies [12]. In light of insights into the immunosuppressive function of TGF-β, the combined blockade of immune checkpoint and TGF-β is under intensive investigation.

TGF-β and PD-L1 act as parallel immunosuppressors through distinct mechanisms. Moreover, it has been revealed that TGF-β could upregulate PD-L1 and PD-1 expression in tumor and T cells, respectively, which serve as additional mechanisms of TGF-β-induced immune suppression [13,14]. In addition, the activation of the TGF-β pathway was linked to a lack of response to PD-1/PD-L1-targeting cancer therapy [15,16], suggesting that inhibition of TGF-β may overcome resistance to anti-PD-1/PD-L1. Furthermore, combining PD-1/PD-L1 inhibition with TGF-β blockade displays synergistic antitumor activity in vivo [15,17,18]. M7824, a recombinant anti-PD-L1/TGF-β bifunctional molecular, demonstrated stronger antitumor efficacy compared to either TGF-β or PD-L1 inhibition [19]. Considering that TGF-β and PD-L1 pathways contribute to suppressing immune response via independent and complementary pathways, simultaneous targeting TGF-β and PD-L1 represents an alternative approach to enhance antitumor immune response and improve PD-1/PD-L1-targeting therapy.

We previously reported the optimization strategies for expressing the bifunctional fusion protein, designated BR102, which simultaneously inhibited TGF-β and PD-L1 signaling [20]. BR102 consists of an anti-PD-L1 antibody fused to the ECD of human TGF-βRII. Three amino acid mutations were introduced into the N-terminus of the TGF-βRII moiety in BR102 to avoid proteolytic degradation and improve druggability [20]. Here, we characterized the preclinical efficacy and safety of BR102 in vitro and in vivo. BR102 can simultaneously disrupt TGF-β and PD-L1 signal and exert greater antitumor efficacy compared with anti-PD-L1 alone. We further demonstrate that BR102 has a good safety profile in an NHP toxicity study. These data suggest that BR102 may provide a promising approach to cancer immunotherapy and improve the efficacy of anti-PD-1/PD-L1.

## 2. Materials and Methods

### 2.1. Antibody Generation and Purification

Anti-PD-L1 antibodies were selected by binding to recombinant human PD-L1 ectodomain in a human library expressed in a phage presentation system. One positive clone, HS636, was obtained and generated on a human IgG1 backbone with N297A mutation to inactivate the Fc-mediated effect function. BR102 was designed by fusing the C-terminus of the HC of HS636 to the N-terminus of the TGF-βRII ectodomain sequence via the (GGGGS)_4_G linker. Amino acid mutations were introduced into the N-terminus of the ectodomain of TGF-βRII to avoid proteolytic degradation. CHO-K1 cells were transfected with vectors encoding HS636 and BR102, respectively. After cell cultures, HS636 and BR102 were purified from cell supernatants by affinity chromatography. Atezolizumab was from Genentech Inc (San Francisco, CA, USA). Human IgG1 isotype control was expressed by Biointron Biological (Taizhou, China).

### 2.2. Cell Lines and Cell Culture

Dendritic cells (DCs) and peripheral blood mononuclear cells (PBMCs) were purchased from AllCells (Shanghai, China). TF-1 cells were from the American Type Culture Collection (Manassas, VA, USA). TGF-β reporter gene HEK-293 cells were purchased from Genomeditech (Shanghai, China). PD-L1 aAPC/CHO-K1 cells and PD-1 Effector cells were purchased from Promega (Madison, WI, USA). Cells were propagated in culture conditions as recommended by the manufacturer.

### 2.3. Antibody Affinity Measurement

The binding affinity of HS636 to PD-L1 was performed on a Biacore X100 instrument (GE Healthcare, Chicago, IL, USA). The anti-human antibody (human antibody capture kit; GE Healthcare) was immobilized on a CM5 sensor chip using the amine coupling kit (GE Healthcare). HS636 (1.0 μg/mL) was captured on the CM5 chip at the rate of 10 μL/min. A dilution of PD-L1-His (R&D Systems, Minneapolis, MN, USA) was injected for 180 s at a flow rate of 30 μL/min. The dissociation phase was monitored for 300 s. The sensor chip was regenerated with 3M MgCl_2_ at a flow rate of 20 μL/min for 60 s. The binding kinetics was recorded and analyzed using Biacore X100 evaluation software.

### 2.4. Binding ELISA Assay

PD-L1 (ACROBiosystems, Beijing, China), TGF-β1 (ACROBiosystems), TGF-β2 (Novoprotein, Shanghai, China), TGF-β3 (R&D Systems), cynomolgus monkey PD-L1 (ACROBiosystems), or TGF-β1 (Sino Biological, Beijing, China) were coated onto high-binding microtiter plates (Corning Inc., Corning, NY, USA) in PBS at 4 °C overnight. The plates were blocked with 5% BSA in PBS and incubated with serially diluted test mAbs. HRP-labeled goat-anti-human IgG (Sigma, St.Louis, MO, USA, 1:5000) was added to the plates and incubated for 1 h at 37 °C. TMB (Huzhou InnoReagents, Huzhou, China) chromogenic reaction was stopped with 1M H_2_SO_4,_ and the absorbance at 450 nm was determined using a Spectramax M5 microplate reader (Molecular Devices, San Jose, CA, USA). A dose response curve was fitted by 4-parameter logistic (4PL) regression (Prism 8; GraphPad, San Diego, CA, USA).

The ability of BR102 to simultaneously recognize PD-L1 and TGF-β1 was also tested by ELISA. BR102 was added to TGF-β1-coated plates, followed by biotinylated PD-L1 (ACROBiosystems) detected with streptavidin-HRP (Abcam, Cambridge, MA, USA, 1:1000). The remaining steps followed the ELISA procedure described above.

### 2.5. Competition ELISA Assay

The blocking ability of HS636 and BR102 was tested in competition ELISA assays. Fc-tagged human PD-1 (ACROBiosystems) was absorbed into high-binding microtiter plates (Corning) at 0.25 µg/mL in PBS at 4 °C overnight. The plates were blocked with 5% BSA in PBST. Biotinylated PD-L1 (ACROBiosystems) and serially diluted HS636 or BR102 were added to the plates. The samples were then incubated with Streptavidin-HRP (Abcam). TMB color development was stopped with 1M H_2_SO_4_, and the absorbance was detected at 450 nm using a Spectramax M5 microplate reader (Molecular Devices). For CD80/PD-L1 blocking assay, Fc-tagged human CD80 (ACROBiosystems) was coated at 0.5 µg/mL, and biotinylated PD-L1 was added at 4 µg/mL.

For the TGF-β1/TGF-βRII blocking assay, varying amounts of BR102 and biotinylated TGF-βRII (ACROBiosystems) were added to 96-well plates pre-coated with TGF-β1 (ACROBiosystems). Bound TGF-βRII was detected by adding streptavidin-HRP (Abcam) followed by TMB. Absorbance at 450 nm was read on a microplate reader (Molecular Devices).

### 2.6. PD-1/PD-L1 Reporter Gene Assay

PD-L1 aAPC/CHO-K1 cells were seeded in 96-well plates at a density of 4 × 10^4^ cells per well. After incubation overnight at 37 °C, PD-1 effector cells and serially diluted HS636 or BR102 were added to the plate and incubated at 37 °C for 6 h. Thereafter, Bio-Lite Luciferase Reagent (Vazyme, Nanjing, China) was added, and the luminescence was measured after 10 min. Data were analyzed using GraphPad Prism software.

### 2.7. TGF-β Reporter Gene Assay

TGF-β reporter gene HEK-293 cells were designed for monitoring the activity of the SMAD signal induced by TGF-β. Cells were seeded at a density of 1.25 × 10^4^ cells per well in 96-well plates and incubated overnight at 37 °C. 0.2 ng/mL TGF-β1 and serially diluted BR102 were added and incubated for 7 h. Bio-Lite Luciferase Reagent (Vazyme) was added, and the luminescence was measured.

### 2.8. TF-1 Cell Proliferation

TF-1 cells were incubated with serially diluted BR102 in the presence of 5 ng/mL IL-4 (Sino Biological) and 0.25 ng/mL TGF-β1 (ACROBiosystems) for 2 days at 37 °C. The proliferation of TF-1 cells was measured by a CellTiter-Glo kit (Promega).

### 2.9. Allogeneic Mixed Lymphocyte Reaction

Mixed lymphocyte reaction (MLR) was utilized to determine T cell activation induced by HS636 and BR102. For HS636, monocytes were isolated from PBMCs using CD14 microbeads (Miltenyi Biotec, Bergisch Gladbach, Germany) and grown in Mo-DC differentiation medium (Miltenyi Biotec) and Mo-DC maturation medium (Miltenyi Biotec) to generate immature dendritic cells (DCs). Human CD4 + T cells isolated from a different PBMC donor were co-cultured with the DCs in a 96-well plate, and indicated antibodies were added. Alternatively, DCs were stimulated with Mitomycin C (50 μg/mL, Selleck, Houston, TX, USA) at 37 °C for 30 min. Subsequently, DCs (5 × 10^3^ cells) were co-cultured with allogeneic PBMCs (1 × 10^5^) in the presence of BR102 or HS636. After 3 days of culture, IFN-γ or IL-2 secretion in cell supernatants were analyzed by ELISA.

### 2.10. Mouse Tumor Xenograft Models

For the human PD-L1 humanized MC38 (MC38/hPD-L1) colorectal syngeneic model, 5 × 10^5^ MC38/hPD-L1 cells (Biocytogen, Beijing, China) were implanted subcutaneously into the right flank of PD-1-humanized C57BL/6 mice (Biocytogen). The mice were randomized into five groups (vehicle control; 1, 3 and 10 mg/kg HS636; 10 mg/kg Atezolizumab; *n* = 10 for each group) when mean tumor volume reached approximately 160 mm^3^. Mice were intraperitoneally injected with vehicle control, Atezolizumab or HS636, every other day for a total of 8 times.

For the HCC827 non–small cell lung cancer model, NCG mice (Gempharmatech, Nanjing, China) were injected subcutaneously in the right flank at day 0 with 5 × 10^6^ HCC827 cells. At day 5, mice were intravenously injected with 1 × 10^7^ PBMCs. HS636 (1.5 mg/kg) or hIgG1 were intraperitoneally injected at days 5, 8, 12, 15, 19, 22 (*n* = 6 for each group).

MC38/hPD-L1 model was used to evaluate the antitumor efficacy of BR102 and HS636. 1 × 10^6^ MC38/hPD-L1 cells were injected subcutaneously into C57BL/6JNifdc mice (Vital River, Beijing, China). The mice were injected intraperitoneally with PBS, HS636 (1 mg/kg), or BR102 (1.22 mg/kg, 3.66 mg/kg) twice a week, beginning when tumors had achieved an average size of 50 mm^3^ (*n* = 8 for each group). For all the models, tumor volume was measured twice per week. The tumor volume (TV) was measured and calculated using the formula: TV (mm^3^) = 1/2 × length × width^2^.

### 2.11. Toxicity Study in Non-Human Primates

The toxicology study in cynomolgus monkeys was conducted at Joinn Laboratories (Taicang, China). Animals (5 animals/gender per group) were administered intravenous (i.v.) infusion of BR102 (15, 50 or 100 mg/kg) or vehicle control (20 mM citrate pH 6.0) once weekly for 4 weeks (D1, D8, D15, D22, and D29). 3 animals/gender/group were euthanized at the end of the dosing period (D30). Following the dosing period, the last 2 animals/gender/group were maintained for a 6-week recovery period and euthanized at the end of the recovery period (D71). In-life evaluations included clinical observations, body weight, food consumption, cardiovascular safety pharmacology evaluations, and clinical pathology. Cytokines, including TNF-a, IFN-γ, IL-2, IL-4, IL-5, and IL-6, were analyzed on a FACSCalibur flow cytometer (BD Biosciences, San Jose, CA, USA) using a cytometric bead array (CBA) Kit (BD Biosciences). Following euthanasia, animals were examined for gross pathology, relative organ weight, and histopathology.

### 2.12. Statistics

Data were presented as mean ± standard error of the mean (SEM) unless otherwise indicated. Statistical significance was analyzed by the Student’s *t*-test. *p* values were considered statistically significant below 0.05 (* *p* < 0.05; ** *p* < 0.01; *** *p* < 0.001; **** *p* < 0.0001).

## 3. Results

### 3.1. Screen and Biological Activity Evaluation of An Anti-PD-L1 Antibody

**Figure 1:**
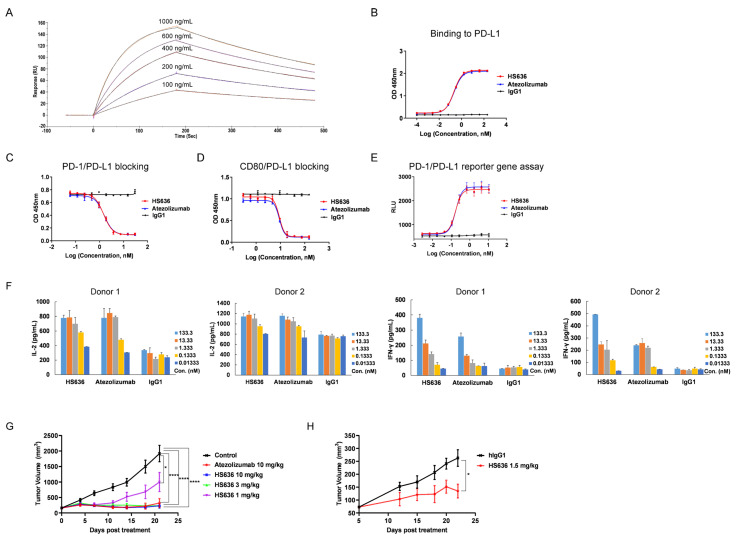
In vitro functional activity and in vivo antitumor efficacy of HS636. (**A**)The affinity of HS636 to PD-L1 was detected by Surface plasmon resonance (SPR). (**B**) The binding activity of HS636 to human PD-L1 was determined by ELISA. The blocking activity of HS636 towards PD-1/PD-L1 interaction (**C**) and CD80/ PD-L1 interaction (**D**) was determined by competition ELISA. (**E**) The bioactivity of HS636 on PD-1/PD-L1 signaling was performed by the PD-1/PD-L1 NFAT reporter gene assay. (**F**) The T cell activation effect of HS636 was determined in MLR assay. CD4 + T cells from 2 donors and allogeneic DCs were co-cultured in the presence of indicated concentrations of HS636 for 3 days, then IL-2 and IFN-γ secretion were quantified by ELISA. (**G**) PD-1 humanized C57BL/6 mice bearing MC38/hPD-L1 tumors were treated with HS636, Atezolizumab, or vehicle control (*n* = 10 for each group). The tumor volume was measured twice per week. (**H**) NCG mice bearing HCC827 tumors were injected with human PBMCs (1 × 10^7^/mouse) and HS636 or isotype control hIgG1 (*n* = 6 for each group). The tumor volume was measured twice per week.

Anti-PD-L1 antibodies were screened to recognize a recombinant human PD-L1 ectodomain fusion protein in a human library expressed in a phage presentation system. One of the positive clones, designated HS636, was obtained and generated on a human IgG1 backbone with N297A mutation to inactivate Fc-mediated effect function, such as ADCC or CDC. HS636 exhibited high affinity to human PD-L1 with a equilibrium dissociation constant (KD) of 4.4 nM, as determined by SPR (Figure 1A). HS636 was also confirmed to bind to PD-L1 by ELISA (Figure 1B). Moreover, HS636 could inhibit PD-L1 binding to both of its receptors, CD80 and PD-1, in a dose-dependent manner (Figure 1C,D). We further evaluated the in vitro potency of HS636. HS636 restored PD-1/PD-L1-dependent NFAT pathway activation in an NFAT-driven luciferase reporter assay (Figure 1E) and induced the release of IL-2 and IFNγ in an MLR assay (Figure 1F). PD-1 humanized C57BL/6 mice xenografted with PD-L1 humanized MC38 colorectal cancer cell line were used to evaluate the in vivo antitumor activity induced by HS636 treatment. Treatment with HS636 significantly inhibited tumor growth compared to the control group (Figure 1G). Similar results were obtained in an NCG mice model using an NSCLC cancer cell line (HCC827 cells) with PBMC cell engraftment plus HS636, which delayed tumor growth (*p* < 0.05) (Figure 1H). We compared HS636 with the FDA-approved anti-PD-L1 antibody, Atezolizumab, concerning efficacy in vitro and in vivo. HS636 displayed similar activities with Atezolizumab (Figure 1B,C,G).

### 3.2. BR102 Could Simultaneously Recognize TGF-β and PD-L1

**Figure 2:**
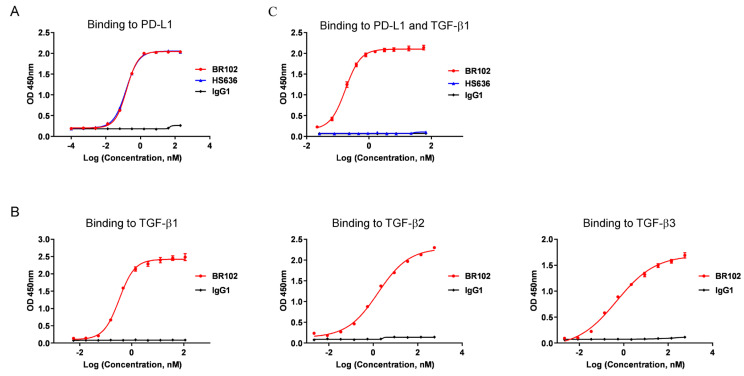
BR102 specifically binds to PD-L1 and TGF-β. The binding of BR102 to PD-L1 (**A**) and various human TGF-β isoforms (β1, β2, and β3) (**B**) was determined by ELISA. (**C**) The simultaneous binding of BR102 to PD-L1 and TGF-β1.

BR102 is a bifunctional fusion protein composed of the anti-PD-L1 antibody HS636, fused at the C-terminus of HC to the ectodomain of TGF-βRII. BR102 bound to PD-L1 with comparable affinity compared to HS636 (Figure 2A). In addition, BR102 could recognize TGF-β1, TGF-β2, and TGF-β3 (Figure 2B), which were the three highly structurally related mammalian TGF-β isoforms. We next evaluate whether the bifunctional molecule BR102 could simultaneously bind to TGF-β1 and PD-L1, and BR102 was confirmed to simultaneously target both the two proteins in an indirect ELISA (Figure 2C).

### 3.3. BR102 Disrupts PD-L1-Mediated Signal and Induces Activation of T Cell

**Figure 3:**
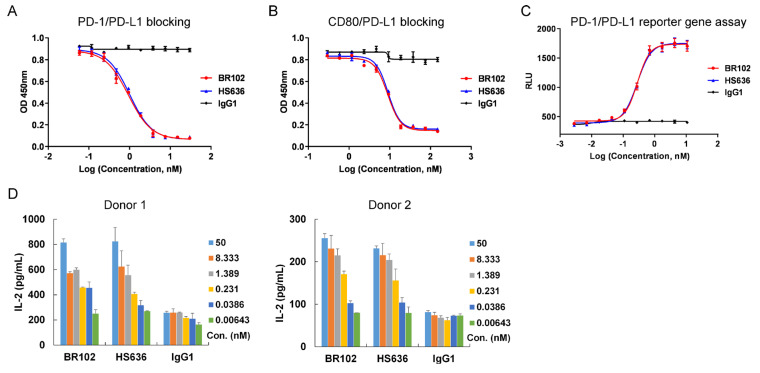
BR102 reversed PD-L1/PD-1 mediated immunosuppression and enhanced T cell activation. BR102 blocked PD-1/PD-L1 interaction (**A**) and CD80/ PD-L1 interaction (**B**) in competition ELISA assays. (**C**) The blockade ability of HS636 on PD-1/PD-L1 signaling was determined by the NFAT reporter gene assay. (**D**) DCs and allogeneic PBMC from 2 donors were co-cultured in the presence of indicated concentrations of BR102 or HS636 for 3 days. IL-2 secretion was analyzed by ELISA.

The effects of BR102 on PD-L1-mediated downstream signal and T cell activation were further characterized. BR102 could block PD-L1 binding to CD80 and PD-1 (Figure 3A,B). Moreover, BR102 relieved the PD-1/PD-L1-induced blockade of NFAT signaling in a luciferase reporter assay (Figure 3C). Then, we evaluated BR102-induced activation of T cells in an MLR assay. BR102 treatment led to the enhancement of IL-2 release (Figure 3D). The in vitro potency of BR102 was similar to that of HS636 (Figure 3A–D), indicating that fusion of TGFβRII ectodomain did not affect the potency of HS636 moiety in BR102. These results suggest that BR102 could block PD-L1-induced immunosuppressive signaling and promote T cell activation.

### 3.4. BR102 Inhibits TGF-β Signal In Vitro

**Figure 4:**
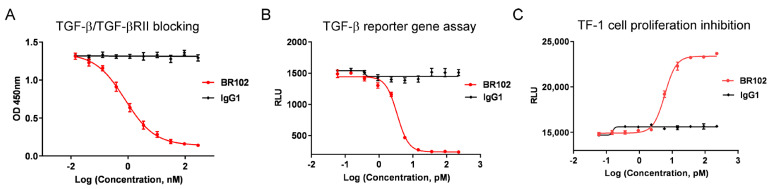
The inhibition effect of BR102 on TGF-β signaling pathway. (**A**) BR102 blocked the interaction of TGF-β1 with TGF-βRII. (**B**) BR102 inhibited TGF-β1-induced SMAD signaling in a TGF-β/SMAD luciferase reporter gene assay. (**C**) BR102 reversed TGF-β1 induced proliferation inhibition of TF-1 cells.

The effects of BR102 on TGF-β-induced signal transduction and cellular function were evaluated. BR102 showed potent antagonism of TGF-β, as determined by inhibiting the interaction between TGF-β and its receptor TGF-βRII (Figure 4A). TGF-β could elicit canonical SMAD pathway upon TGF-β ligand binding with TGF-βRII. The blocking activity of BR102 on TGF-β-mediated SMAD signal was then evaluated using a TGF-β/SMAD luciferase reporter assay, and our data showed that BR102 inhibited SMAD signaling mediated by TGF-β in a dose-dependent manner (Figure 4B). In addition, BR102 also relieved TGF-β-induced growth inhibition in a human erythroleukemic cell line, TF-1 (Figure 4C). The above results suggest that BR102 could act as a potent TGF-β inhibitor.

### 3.5. BR102 Treatment Inhibits Tumor Growth In Vivo

**Figure 5:**
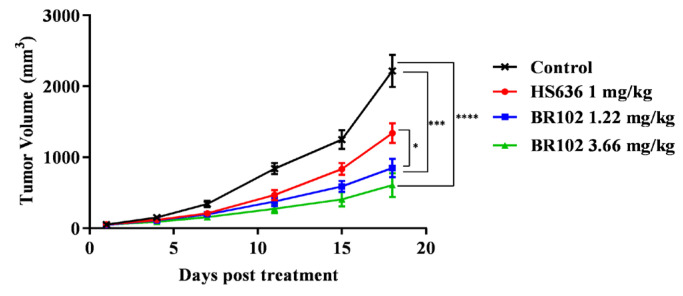
BR102 inhibited tumor growth in a murine tumor model. C57BL/6JNifdc mice were subcutaneously injected with MC38/hPD-L1 cells. When tumor volumes reached 50 mm^3^, mice were treated with PBS, HS636, or BR102 (*n* = 8 for each group). The tumor volume was measured twice per week.

We next evaluated the in vivo efficacy of BR102 in a syngeneic mice model. C57BL/6JNifdc mice implanted subcutaneously with the colon adenocarcinoma MC38/hPD-L1 cell line were assigned to treatment with either PBS (vehicle) or BR102, and the anti-PD-L1 mAb HS636 was included for comparison. Compared with the control group, mice receiving intraperitoneal administration of BR102 showed significant tumor growth inhibition (Figure 5, Figure S2). Moreover, BR102 led to a more potent inhibition of tumor growth compared to the equimolar dose of HS636 (Figure 5). No significant weight loss was observed in mice treated with BR102 (Figure S2).

### 3.6. BR102 Shows A Favorable Safety Profile In Vivo

**Figure 6:**
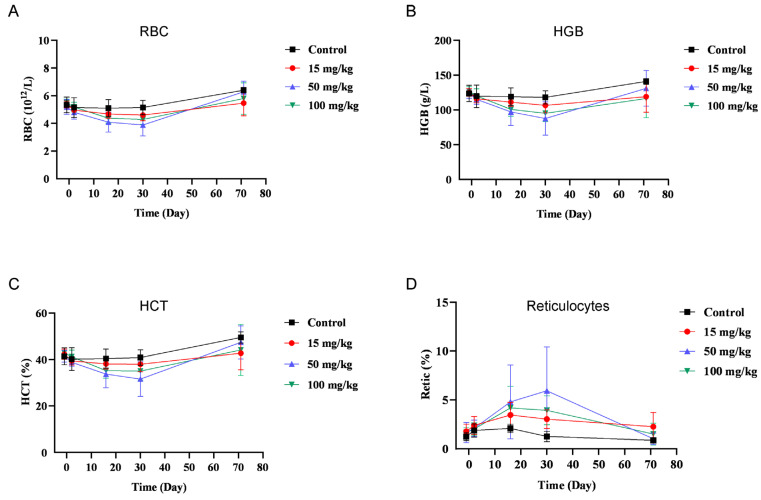
Treatment with BR102 is well tolerated in non-human primates. BR102 was administered to cynomolgus monkeys at 15, 50, and 100 mg/kg (five males and five females for each group) once weekly for a total of 4 weeks. Peripheral blood was collected for hematology during the pre-dose phase; on Days 2 and 16 of the dosing phases; and on Days 30 and 71 of the recovery periods. Hematology parameters, including RBC (**A**), HGB (**B**), HCT (**C**), and reticulocytes (**D**) were measured. Values are presented as mean ± SD.

The safety concerns regarding anti-TGF-β therapies limit their therapy window and challenge the clinical development of TGF-β inhibitors. It was reported that administration of a neutralizing pan-TGF-β antibody in cynomolgus monkeys resulted in adverse effects, including generalized bleeding, cardiovascular toxicity, pathologic changes in the bone, and even mortality [21]. We next investigated whether BR102 would display an improved safety profile. BR102 could bind to cynomolgus TGF-β and PD-L1 (Figure S1), confirming that cynomolgus monkey is a relevant species for in vivo safety study of BR102. A repeat-dose toxicity study of BR102 in cynomolgus monkeys was performed. BR102 was well tolerated at all dose levels tested (15, 50, or 100 mg/kg). The administration of BR102 led to a minimal to moderate decrease in red blood cells (RBCs), hemoglobin (HGB), and hematocrit (HCT), and a corresponding increase in reticulocytes (Figure 6). Importantly, the levels of RBCs, HGB, HCT, and reticulocytes returned to baseline values by the end of the recovery period (Figure 6). No other BR102-related effects were detected on hematology parameters. BR102 treatment did not elicit cardiovascular toxicity, observed with pan-TGF-β antibody or TGF-βR small molecule inhibitors in animal studies [21,22]. There were no BR102-related changes in clinical observations, body weight, food consumption, clinical chemistry parameters, coagulation parameters, gross pathology, relative organ weights, or histopathology. No changes in cytokine release were noted. These data suggest that BR102 has a favorable safety profile in vivo.

## 4. Discussion

Poor response and resistance are the main challenges for PD-1/PD-L1-targeted immunotherapy. Other immunosuppressive regulators within TME may promote immune escape and suppress anticancer immune response [23]. TGF-β is secreted by multiple cell types in TME, which includes tumor cells, T cells, macrophages, and MDSCs. TGF-β exerts immune suppression and promotes tumor progression through its effects on both the innate and adaptive immune systems. It was shown that TGF-β could promote the expansion of Treg cells and inhibit the function of cytotoxic T cells and DCs [24,25]. In addition, TGF-β could also impair NK function and drive myeloid cell-mediated tumor metastasis [26,27,28]. Moreover, TME-derived TGF-β could augment the expression of PD-1 in tumor-infiltrating lymphocytes, which causes CD8+ T cell suppression and immune resistance [14]. These indicated that TGF-β functions as a critical immune-suppressive cytokine in TME and may engage in crosstalk with PD-1/PD-L1 signal to promote immune escape. These provide a biological rationale for simultaneously targeting PD-L1 and TGF-β immunosuppressive signaling pathway, which could enhance antitumor immunity and overcome resistance to anti-PD-1/PD-L1 therapies.

We developed and characterized the novel bifunctional protein BR102, composed of an anti-PD-L1 antibody (HS636) fused to the ECD of human TGF-βRII. Amino acid mutations were introduced into the N-terminus of the ectodomain of TGF-βRII to avoid proteolytic degradation [20]. BR102 inhibits the binding of PD-L1 to both of its receptors, CD80 and PD-1. Furthermore, BR102 disrupts PD-L1-mediated downstream signaling and induces activation of T cells. The biological activity of BR102 is comparable to that of HS636, indicating that the fusion of TGF-βRII ECD does not affect the efficacy of the anti-PD-L1 antibody moiety. We found that the TGF-βRII moiety of BR102 could bind to the three TGF-β isoforms, TGF-β1, TGF-β2, and TGF-β3, and inhibit TGF-β-mediated signaling. Moreover, BR102 displays more potent antitumor activity compared to HS636 alone. Disruption of both TGF-β and PD-L1 signaling may contribute to the antitumor efficacy of BR102. These results indicate that BR102 could deliver the therapeutic benefit of simultaneously targeting TGF-β and PD-L1 signaling and support further exploiting BR102 as a novel therapy for advanced malignancies. In addition, it was shown that TGF-β-induced immunosuppression contributed to resistance to multiple antitumor treatments, such as radiotherapy, chemotherapy, immunotherapy, and targeted therapy [29]. Consequently, BR102 has the potential to be combined with these therapies to overcome therapy resistance and enhance treatment efficacy in cancer patients.

Besides BR102, other anti-PD-L1/TGF-β bifunctional proteins have been developed, including M7824 and YM101 [19,30]. Both BR102 and M7824 consist of an anti-PD-L1 antibody fused to the ECD of human TGF-βRII, while YM101 is a bispecific antibody developed with the Check-BODY™ platform [19,30]. Compared with M7824, BR102 has three amino acid mutations in the N-terminus of the ECD of TGF-βRII. We previously reported that the introduced amino acid mutations in BR102 could effectively decrease proteolytic degradation and improve druggability [20]. The anti-PD-L1 moieties of BR102 and M7824 are based on HS636 and avelumab, respectively. Although a head-to-head comparison of HS636 and avelumab was not conducted, HS636 displays similar in vitro and in vivo efficacy with another approved-anti-PD-L1, Atezolizumab. This supports that HS636 is a potent PD-L1 antagonist and HS636 is suitable for use as the anti-PD-L1 moiety of BR102. The anti-TGF-β moieties of BR102 and M7824 are based on the ECD of human TGF-βRII, designed as a trap for TGF-β, and the anti-TGF-β moiety of YM101 is based on a TGF-β antibody GC1008 [19,30]. However, the three anti-PD-L1/TGF-β bifunctional proteins could bind all three TGF-β isoforms (TGF-β1, TGF-β2, and TGF-β3). Moreover, BR102, M7824, and YM101 exhibit potent antitumor activity in syngeneic mouse models. Studies revealed that M7824 and YM101 promoted the immune-supportive TME by increased T cell infiltration into tumors, induction of an enhanced DCs density, and polarization of macrophages [19,30]. Further studies are needed to evaluate the effect of BR102 on TME regulation. Regarding safety, M7824 shows a manageable safety profile in clinical trials [31,32], and our data in the preclinical toxicity study also support the favorable safety profile of BR102. No YM101-related safety data have been disclosed or published. The molecular structure, specificity, and affinity of the anti-PD-L1 and anti-TGF-β moieties, may play key roles in determining the efficacy and safety of the anti-PD-L1/TGF-β bifunctional proteins.

Due to the accumulated evidence about the implication of TGF-β in tumor progression, various strategies, including antibodies against TGF-β or TGF-βR, ligand traps, TGF-βRI inhibitors, and antisense oligonucleotides have been explored to target TGF-β signaling and are being evaluated in clinical trials. However, only a minor clinical benefit and limited success using these TGF-β-targeting therapies were observed in the clinical setting. Moreover, adverse effects are another issue that challenges the clinical development of TGF-β-targeting therapies. It has been shown that blocking the TGF-β signaling with antibodies or TGF-βR kinase inhibitors resulted in cardiovascular toxicity in animals [21,22]. The cardiac side effects may be mitigated by employing an intermittent dosing schedule in human clinical trials using galunisertib, a TGF-βRI kinase inhibitor [33,34,35]. This indicated that optimal dosing regimens are needed to decrease toxicity for exploring TGF-β-targeted therapies. In addition, reversible cutaneous keratoacanthomas and squamous-cell carcinomas were observed in patients administrated with fresolimumab (GC1008), a TGF-β antibody [36]. In a phase I study evaluating the safety of an anti-TGF-βRII antibody (LY3022859) to treat patients with solid tumors, the maximum tolerated dose was not defined because of negative symptoms, such as uncontrolled cytokine release, despite prophylaxis [37]. The challenges for clinical development of TGF-β pathway antagonists may be largely due to the pleiotropic effect and dual function of TGF-β. TGF-β plays a significant role in embryonic development and maintenance of adult tissue homeostasis by transmitting its canonical and non-canonical signals. TGF-β functions as a tumor suppressor in the early stages and as a tumor promoter in the late stages. Mutations of various components of TGF-β signals may prompt the conversion of the TGF-β role in different tumor stages [38,39]. The pleiotropic nature of TGF-β restricts the clinical development of pharmacological TGF-β-targeted agents, which can affect normal tissue and lead to unwanted side effects. Considering the high PD-L1 expression within TME, the bifunctional anti-PD-L1/TGF-βRII fusion protein BR102 could be anticipated to lead to a more tumor-targeted inhibition of TGF-β within the TME and reduce safety concerns associated with some TGF-β targeted therapies. In the preclinical NHP toxicity study, BR102 displays a favorable safety profile. BR102 treatment does not cause cardiovascular toxicity and does not induce enhanced cytokine release. The red cell changes induced by BR102 are reversible. BR102 is being evaluated in clinical trials to test the safety and preliminary efficacy in advanced-stage cancer patients.

BR102 consists of the TGF-βRII moiety, which recognizes TGF-β1, TGF-β2, and TGF-β3. BR102 would likely block signals downstream of TGF-βR mediated by all three TGF-β isoforms. These isoforms are highly similar in sequence and structure, but show differential expression patterns in vivo. Studies reveal that TGF-β1 is the most prevalent member responsible for TGF-β pathway activity in many human tumor types. Selective TGF-β1 blocking could overcome resistance to ICIs in a mouse tumor model [40]. Although TGF-β2 and TGF-β3 are less frequently expressed in tumors, they may also be implicated in the progression of certain cancers. For example, TGF-β2 is overexpressed in glioblastoma and has been associated with poor clinical outcomes [41], and expression of TGF-β3 is shown to promote head and neck cancer growth and metastasis [42]. However, the role of TGF-β2 and TGF-β3 in tumor development must be better determined. Some Pan-TGF-β inhibitors that target all three TGF-β isoforms have been developed and are evaluated in clinical trials. In addition, isoform-selective inhibitors are also identified and selected for clinical evaluation, with the rational that that selectively targeting the cancer-relevant TGF-β pathway may avoid the side effects of broad TGF-β inhibition and benefit most from the TGF-β targeting. A specifically anti-TGF-β1 neutralizing antibody did not show clinical efficacy in the clinical evaluation as a monotherapy [43]. AVID200, an engineered TGF-β ligand trap that selectively binds and neutralizes TGF-β1 and TGF-β3, is currently in phase 1 trial for patients with advanced solid tumors (NCT03834662). NIS793 is an anti-TGF-β1/2 antibody combined with chemotherapy in a phase 2 trial patients with solid metastatic tumors (NCT04390763). More clinical outcomes are needed to evaluate whether these isoform-selective inhibitors could lead to a more favorable clinical benefit. In addition, considering that the PD-L1 and TGF-β are nonredundant pathways mediating immunosuppressive activity within TME, it is suggestive that BR102, the bifunctional molecular that simultaneously targets PD-L1 and TGF-β, may provide an alternative strategy to improve anti-TGF-β therapies. Furthermore, it is crucial to uncover biomarkers for defining patients who will benefit from TGF-β-targeted treatment. Several reports indicate that high expression of TGF-β target genes and mesenchymal subtypes correlate with poor prognosis in patients with many different cancer types, including CRC, hepatocellular carcinoma, and lung cancer [44,45,46]. A better understanding of the underlying mechanisms by which TGF-β signaling regulates normal and malignant processes will facilitate appropriate patient selection in clinical trials and advance the development of TGF-β antagonists.

## 5. Conclusions

BR102 is a novel bifunctional molecule that could simultaneously target the two immunosuppressors TGF-β and PD-L1. BR102 shows a higher antitumor efficacy than the anti-PD-L1 antibody, as well as a more favorable safety profile compared with some TGF-β-target therapies. The data support further development of BR102 for treating various tumor types.

## 6. Patents

Patent applications related to this work have been filed by BioRay (WO/2022/063114).

## Data Availability

All data in the current study are available from the corresponding author upon reasonable request.

## Supplementary Materials

The following supporting information can be downloaded at: https://www.mdpi.com/article/10.3390/cancers14194964/s1, Figure S1: Binding of BR102 to cynomolgus monkey PD-L1 and TGF-β1 were assessed by ELISA. Figure S2: Tumor weights (A), tumor images (B), and body weights (C) of MC38/hPD-L1-bearing mice treated with HS636 or BR102.
