# Supplementary material for: Development of the Novel Bifunctional Fusion Protein BR102 That Simultaneously Targets PD-L1 and TGF-β for Anticancer Immunotherapy
Source: Cancers (Basel). 2022 Oct 10;14(19):4964. doi: 10.3390/cancers14194964 (PMC9562016; doi:10.3390/cancers14194964)
Supplement: Supplementary file 1 [file cancers-14-04964-s001.zip › cancers-1959133-supplementary.pdf]

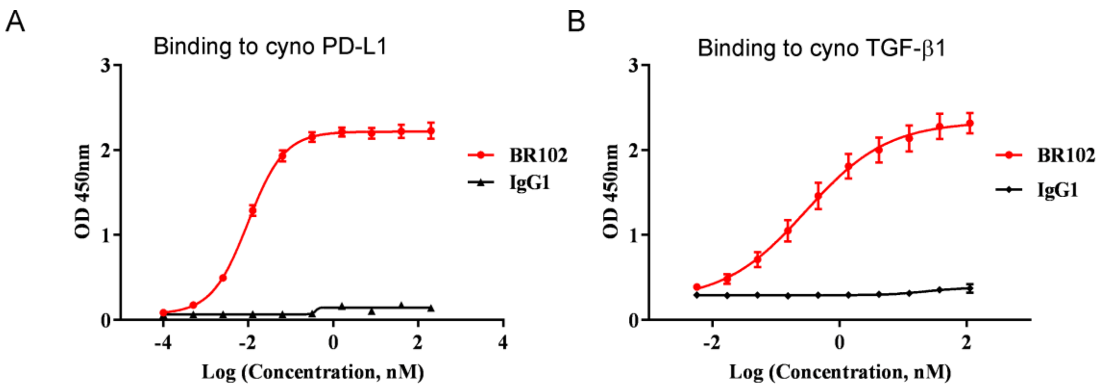

Figure S1. Binding of BR102 to cynomolgus monkey PD-L1 and TGF-β1 were assessed by ELISA.

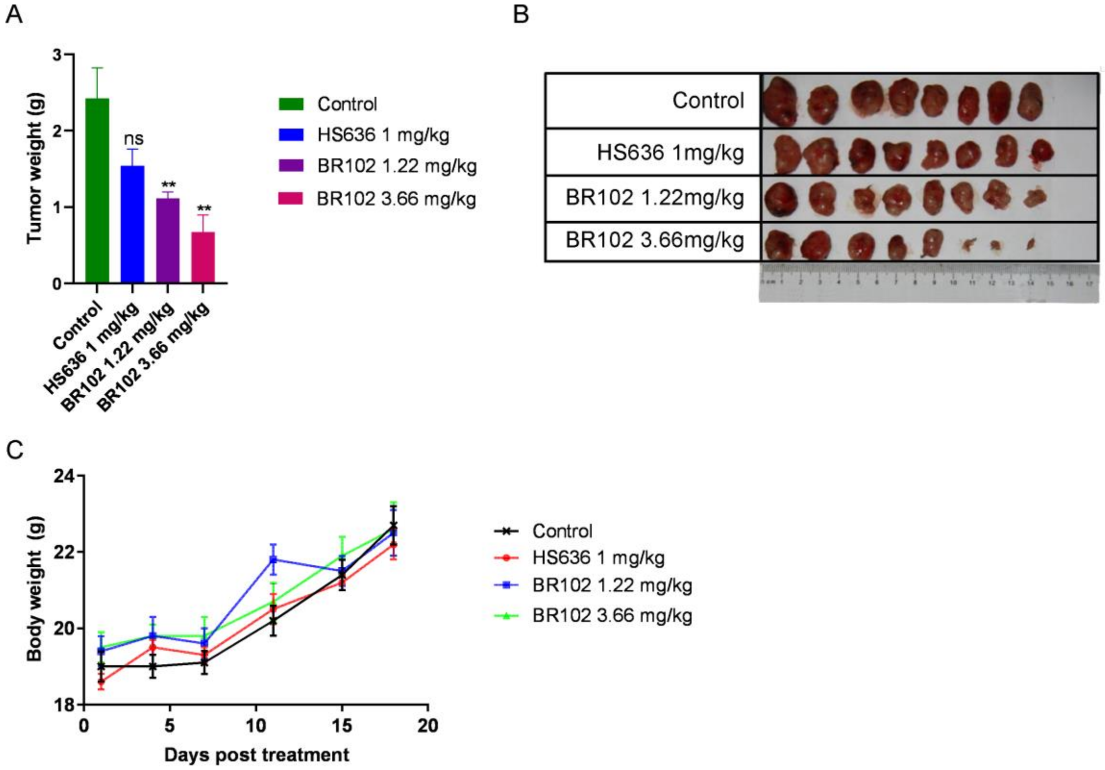

Figure S2. Tumor weights (**A**), tumor images (**B**) and body weights (**C**) of MC38/hPD-L1-bearing mice treated with HS636 or BR102. *Indicate statistical differences compared with the control group. ** $p < 0.01$; ns, not significant.
